# Sociodemographic disparities in primary care management of back pain in children and young people: a population-based cohort study

**DOI:** 10.1007/s00586-026-09867-4

**Published:** 2026-03-14

**Authors:** Kayleigh J Mason, Kelvin P Jordan, James Bailey, Joanne Protheroe, Martin J Thomas, Faraz Mughal, Anna Saxne Jöud, Sue Jowett, Kym I E Snell, Kate M Dunn

**Affiliations:** 1 Primary Care Centre Versus Arthritis, School of Medicine, Keele University, Newcastle-under-Lyme, UK; 2 Haywood Academic Rheumatology Centre, Midlands Partnership University NHS Foundation Trust, Haywood Community Hospital, Stoke-on-Trent, UK; 3 Department of Applied Health Sciences, School of Health Sciences, College of Medicine and Health, University of Birmingham, Birmingham, UK; 4 Oxford Primary Care Clinical Trials Unit, Nuffield Department of Primary Care Health Sciences, University of Oxford, Oxford, United Kingdom; 5 Orthopedics, Department of Clinical Sciences Lund, Lund University, Lund, Sweden; 6 Occupational and Environmental Medicine, Department of Laboratory Medicine, Lund University, Lund, Sweden; 7 Department of Applied Health Sciences, School of Health Sciences, College of Medicine and Health, University of Birmingham, Birmingham, UK; 8 NIHR Birmingham Biomedical Research Centre, Birmingham, UK

**Keywords:** Paediatric, Epidemiology, Primary care, Back pain, Management, Analgesia, Referrals

## Abstract

**Background:**

Research on the primary care management of back pain in children and young people (CYP) is scarce. Sociodemographic factors may influence presentation and management, potentially contributing to disparities in care and outcomes. This study aimed to describe the management of back pain in CYP and examine associations with sociodemographic characteristics.

**Methods:**

Data were extracted from the Clinical Practice Research Datalink (CPRD) Aurum for 425,000 randomly sampled CYP aged 8–18 years with a new musculoskeletal pain episode between 2005 and 2021. Analgesia prescriptions, referrals and imaging within 6 months of an index consultation for back pain were extracted. Adjusted risk ratios (aRR) were calculated for age, gender, deprivation, ethnicity, region and index year.

**Results:**

Of 51,335 CYP presenting with back pain, 78% had symptom-based codes for back pain or low back pain, and 12% had more specific diagnostic codes such as spinal deformities. 19% patients reconsulted for back pain in the 6 months following the index date. Analgesia was prescribed to 36% with higher prescribing rates among older CYP and those from more deprived areas (aRR most vs. least deprived 1.64; 95% CI 1.56, 1.72). Referrals (28%) and imaging (13%) were more common in older adolescents but less frequent among more deprived and minority ethnic groups. Prescribing declined over time (46% in 2005–2009 to 16% in 2020–2021), whereas referrals increased (17% to 40%).

**Conclusions:**

Back pain in CYP often recurs and is primarily managed with analgesia and referrals. Socioeconomic and ethnic disparities in management highlight the need for equitable access to specialist care and consistent, evidence-based approaches.

**Supplementary Information:**

The online version contains supplementary material available at https://doi.org/10.1007/s00586-026-09867-4.

## Introduction

Back pain is common among adults and is a key reason for disability, time off work and healthcare seeking [1–4]. One of the most important predictors of back pain onset and progression is a previous history of back pain, even among younger adults [5–7] and among primary care patients considered to have new onset symptoms, 75% have a history of back pain [8]. There is evidence that pain in childhood increases the risk of chronic musculoskeletal pain among adults [9–11], meaning that childhood is a period of importance for understanding back pain across the lifecourse.

Children and young people (CYP) already have a lifetime prevalence of back pain of approximately 50% [12, 13], with a one-month prevalence of approximately 25% [14, 15], and chronic back pain (≥ 3 months duration) is present in approximately 11% [16]. Back and neck pain is among the top 10 causes of disability globally among 10–14-year-olds, rising to fourth place among 15–19-year-olds, before becoming the leading cause among 25–29-year-olds [4]. The incidence of back pain clearly increases with age throughout childhood and adolescence [17–20]. with levels likely reaching those observed in adulthood in the early to mid–20s [21]. Our previous study revealed that approximately 1% of CYP aged 8–18 seek healthcare for back pain each year, with the figure rising with age to become the most common musculoskeletal healthcare consultation among females aged 16–18 years and second only to knee pain for males aged 16–18 years [22].

While there is evidence about paediatric back pain in community settings, information on healthcare seeking and management in primary care or other first contact settings is lacking, even though this is where most management will occur. Furthermore, we are aware of only one back pain clinical guideline, specifically for CYP [23] and UK guidelines for adults exclude those under 16 years of age [24]. This gap is even more significant considering concerns about opioid prescribing and inequities in access to specialist care [25, 26].

In addition, socioeconomic and demographic factors are known to influence the presentation and management of health conditions in CYP [27]. Children from more deprived neighbourhoods or ethnic minority groups may face barriers to timely diagnosis, access to physiotherapy, or imaging services, potentially perpetuating disparities in care and outcomes [28–30]. However, few studies have examined how such characteristics affect clinical management in the context of paediatric back pain. We hypothesise that individuals residing in more deprived areas and minority ethnic groups have different patterns of presentation and management, potentially contributing to disparities in care and outcomes.

These gaps and the need for epidemiological evidence describing the size and impact of musculoskeletal health have been suggested by calls to action [11, 31]. To address this, we used a large, representative primary care dataset to describe the management of musculoskeletal back pain in CYP aged 8–18 years and determine associations with sociodemographic characteristics.

## Methods

The study used the UK Clinical Practice Research Datalink (CPRD) Aurum, a database of anonymised primary care electronic health records of over 41 million patients from over 1,400 general practices via EMIS Web^®^ software, which currently covers 20% of the UK population [32, 33]. CPRD is representative of the UK population and the use of linked data (patient-level Index of Multiple Deprivation data and CPRD Aurum Ethnicity Record) provides representative distributions on neighbourhood-level deprivation and ethnicity compared to national data on the UK population [34, 35].

The study was approved by the CPRD Research Data Governance (ref 22_002318) and used the May 2022 release [32]. The approved protocol was made available to the reviewers.

### Study population

CYP aged 8–18 years with a recorded primary care consultation for musculoskeletal pain between 2005 and 2021, no consultation for musculoskeletal pain in the previous 12 months, and with at least 24 months prior registration at their general practice before 1st January of each calendar year were eligible for inclusion in the study population. Given the high incidence of musculoskeletal pain [22], a random sample of 25,000 CYP per year who fulfilled the study population criteria were selected, resulting in a total sample size of 425,000. CYP could only be included once in the study population. Selected CYP were followed up for six months from the date of their primary care consultation for back pain (index date) by consensus of study team, including 2 GPs, to allow sufficient time for a diagnosis, analgesia prescribing, referral to specialist care and/or imaging to occur and be captured in the electronic health record. Musculoskeletal pain consultations were categorised by pain site. From the final sample, we included all those with recorded back pain on the index date in this analysis.

In UK primary care, the Read Code system was used to record diagnoses and symptoms up to 2018, with SNOMED CT codes used from 2018. The Read and SNOMED CT code lists for musculoskeletal pain were derived from previous studies [36, 37] and reviewed and finalised by the consensus of two academic general practitioners and a specialist rheumatologist and musculoskeletal physiotherapist. The final musculoskeletal code list is available at https://doi.org/10.21252/eye3-tq63, with the code list for back pain in the Supplementary Materials, Table S1.

Some of the included codes may not always be associated with pain in childhood (e.g., lordosis, kyphosis, congenital abnormalities) but were included as likely to be causing discomfort or impacting on daily life if recorded as a reason for consulting primary care.

### Outcomes

Recording of trauma on the index date and of specific diagnoses on the index date or in the following 6 months were determined. The management outcomes included referrals, imaging, and analgesic prescriptions for musculoskeletal conditions on the date of the index consultation and within the following 6 months (referred to as on/within 6 months of the index date).

Referrals included musculoskeletal services, paediatrics, pain clinics, physiotherapy, rheumatology, and orthopaedics. Referral destinations to be included were agreed between the study team and a patient and public involvement (PPI) group of CYP with experience of musculoskeletal pain and their parents.

An adult analgesia classification [38] was modified for CYP through the agreement of GPs/researchers with primary care EHR expertise to ensure relevance to this age group. This classification categorises medications into groups (basic analgesia, weak to very strong opioids, oral NSAIDs, gabapentinoids/amitriptyline, and duloxetine). Basic analgesia included paracetamol, aspirin, topical NSAIDs, and low–dose (≤ 400 mg) ibuprofen.

We also ascertained further musculoskeletal consultations during the six–month follow–up.

### Covariates

Covariates included age at index consultation (grouped into 8–11, 12–15, and 16–18 years; age groups informed by the PPI group), gender, ethnicity, geographical region, neighbourhood deprivation, and index year (2005–2021).

Gender is the term used by CPRD Aurum as individuals can request to have their gender identity recorded instead of biological sex on their primary care record (following Gender Recognition Act 2004 [39]).

Ethnicity was defined using a code list categorised into Bangladeshi, Black African, Black Caribbean, Chinese, Indian, Other Asian, Other, Pakistani, and White ethnic groups. The code list is available at https://doi.org/10.21252/eye3-tq63.

Geographical region is a field provided by CPRD. We have grouped GP practices into London, Midlands/East England, North England, South England, and Northern Ireland regions.

Neighbourhood deprivation was based on the English Index of Multiple Deprivation (IMD) 2019, a composite measure covering seven domains (health deprivation and disability; barriers to housing and services; employment; income; education, skills and training; crime; living environment) and is determined at small geographical area level for average resident population size of 1500 [35]. CYP were categorised into 5 groups from least to most deprived on the basis of the national quintile rank.

### Sample size

Based on previous studies in CYP, we expected approximately 13–15% of the 425,000 sample to have consulted for back pain [2, 40]. A resulting sample size of approximately 60,000 allows determination of the percentage with each outcome with a 95% confidence interval (CI) of ± 0.4%, assuming an estimated percentage of 50%. The sample size calculation was performed using Power Analysis and Sample Size (PASS) Software (2022; NCSS, LLC. Kaysville, Utah, USA, ncss.com/software/pass).

### Analysis

Frequencies and percentages for each outcome by the covariates were determined. Robust Poisson regression was used to determine the independent associations of the covariates (age; gender; ethnicity; region; deprivation; index year) with analgesia prescription, referral, and imaging on the index date or the following six months. Estimates of associations are presented as risk ratios (RRs) and adjusted RRs (aRRs) with 95% CIs. All models included robust standard errors clustered at the practice level. Individuals with missing data for neighbourhood-level deprivation and ethnicity were included in analysis models in a “missing” category for each variable.

As a sensitivity analysis, associations of covariates with management were repeated for the period 2015–2019 to remove any impact of changing trends over time and of the COVID–19 pandemic.

Data management and statistical analyses were performed in STATA version 18 (StataCorp, USA).

## Results

There were 1,665,708 CYP who fulfilled our inclusion criteria. The random sample of 425,000 matched the full cohort on gender (52% male) and age (median 13 years, IQR 11, 16) distributions [22]. A total of 51,335 (12%) CYP presented with back pain on the index date and formed the study population (Table 1); 78% had symptom-based codes for back pain or low back pain, 12% had more specific diagnostic codes such as spinal deformities (Supplementary Materials, Table S1). 5% of CYP with back pain had a trauma code recorded on the index date (highest in males aged 8–11), and 6,146 (12%) had a diagnosis recorded on the index date or in the following 6 months, with spinal deformity (scoliosis, kyphosis, lordosis) being the most common (61% of all diagnoses; Table 1). One in four CYP reconsulted for musculoskeletal pain within six months of the index date, with most of these patients reconsulting for back pain.

**Table 1:** Type of back pain and further consultation by age and gender

		Females			Males		
	Total	Age 8–11	Age 12–15	Age 16–18	Age 8–11	Age 12–15	Age 16–18
Patients, n	51,335	4,484	11,728	13,096	3,317	9,085	9,625
MSK trauma recorded on index date, *n* (%)	2,427 (5)	288 (6)	438 (4)	390 (3)	268 (8)	571 (6)	472 (5)
MSK diagnosis recorded on index date, *n* (%)	4,406 (9)	408 (9)	1,334 (11)	968 (7)	256 (8)	722 (8)	718 (7)
MSK diagnosis recorded: Days 1–183^a^, *n* (%)	1,740 (3)	132 (3)	431 (4)	437 (4)	94 (3)	355 (4)	291 (3)
MSK diagnosis recorded, n (%)							
*Scoliosis/Kyphosis/Lordosis*	3,762 (7)	381 (8)	1,265 (11)	663 (5)	243 (7)	650 (7)	560 (6)
*Disc herniation/Sciatica/Spondylosis*	582 (1)	19 (0)	84 (0)	272 (2)	8 (0)	65 (1)	134 (1)
*Other*	1,802 (4)	140 (3)	416 (4)	470 (4)	99 (3)	362 (4)	315 (3)
Further MSK Consultation: Days 1–183^a^, *n* (%)	12,877 (25)	973 (22)	3,167 (27)	3,532 (27)	666 (20)	2,175 (24)	2,364 (25)
*Back pain*	9,541 (19)	674 (15)	2,412 (21)	2,714 (21)	450 (14)	1,486 (16)	1,805 (19)
*Other MSK pain* ^*b*^	3,336 (6)	299 (7)	755 (6)	818 (6)	216 (7)	689 (8)	559 (6)

^a^ after index date; ^b^ consulted for pain at any other body site, excluding back pain

Management is described in Tables 2, 18 and 657 (36%) CYP had an analgesic prescribed; this was typically a basic analgesic, although the use of NSAIDs and opioids increased in CYP aged 16–18 years (NSAIDs: 18% females, 14% males; opioids: 15% females, 10% males). Gabapentinoids/amitriptyline and duloxetine were rarely prescribed. 28% of CYP were referred onwards, with referrals to physiotherapy, orthopaedics, and paediatrics being the most common; referrals to physiotherapy increased with age, whereas referrals to paediatrics decreased with increasing age. 13% had imaging data requested; there was no clear pattern with age or gender.

**Fig. 1:**
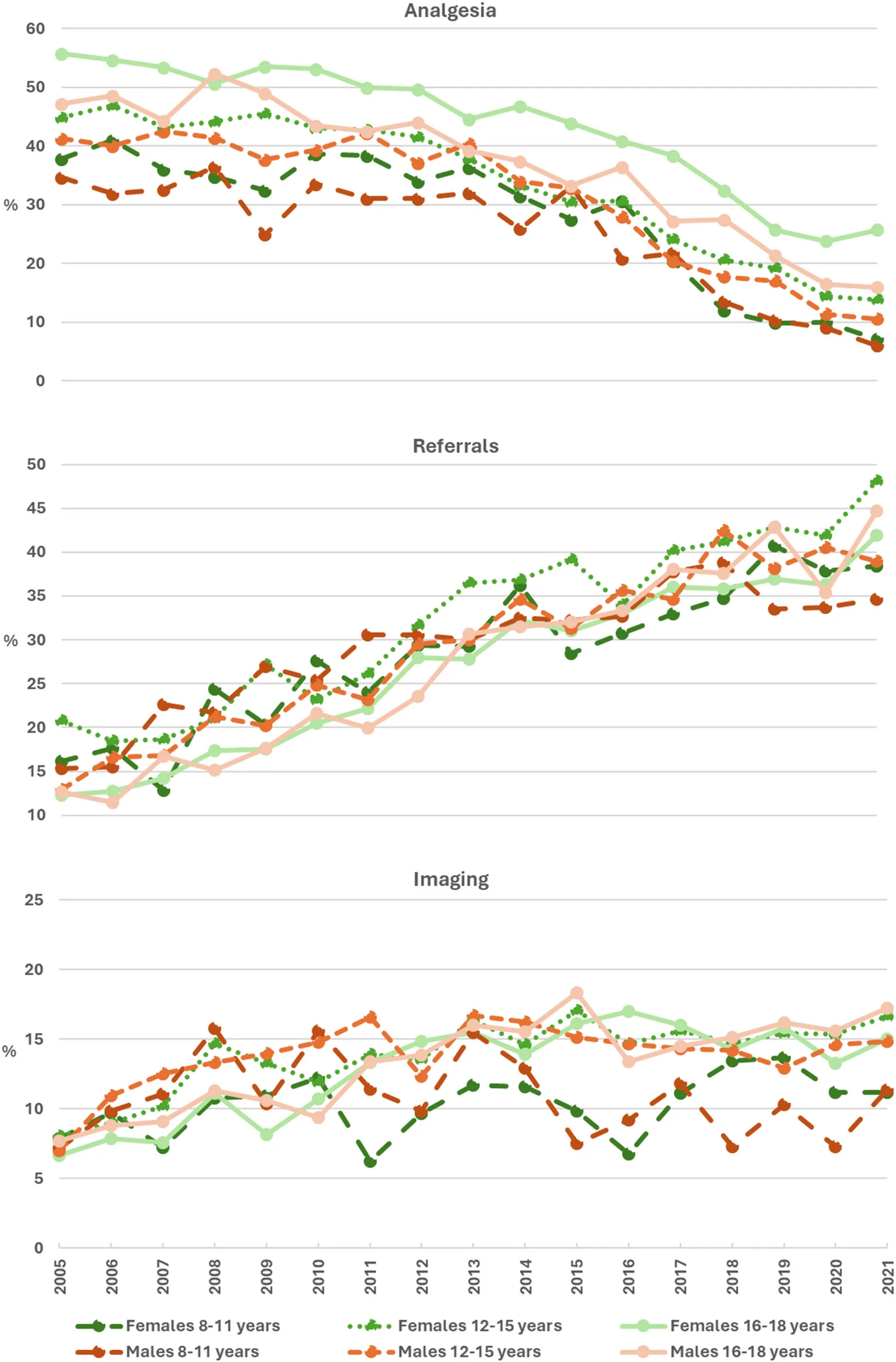
Trends in analgesia prescribing, referrals and imaging on/within 6 months of the index date by age and gender

Prescribing fell considerably over time from 46% of CYP prescribed analgesia in 2005–2009 to 16% in 2020–2021 (Fig. 1 by age and gender; Supplementary Materials, Figure S1 by type of analgesia). In contrast, referrals increased substantially (17% of CYP in 2005–2009 to 40% in 2020–2021; Supplementary Materials, Figure S1).

**Table 2:** Management on/within 6 months of the index date by age and gender

		Females			Males		
	Total	Age 8–11	Age 12–15	Age 16–18	Age 8–11	Age 12–15	Age 16–18
Patients, *n*	51,335	4,484	11,728	13,096	3,317	9,085	9,625
Analgesia prescribed within 6 months^a^, *n* (%)	18,657 (36)	1,268 (28)	4,029 (34)	5,869 (45)	843 (25)	2,932 (32)	3,716 (39)
*Basic*	12,855 (25)	1,240 (28)	3,171 (27)	2,948 (23)	820 (25)	2,556 (28)	2,120 (22)
*Any opioid*	3,963 (8)	30 (< 1)	617 (5)	2,027 (15)	25 (< 1)	336 (4)	928 (10)
*Weak Opioid*	2,248 (4)	11 (< 1)	381 (3)	1,134 (9)	11 (< 1)	208 (2)	503 (5)
*Moderate Opioid*	812 (2)	12 (< 1)	139 (1)	422 (3)	8 (< 1)	84 (1)	147 (2)
*Strong/Very Strong Opioid*	1,181 (2)	9 (< 1)	130 (1)	658 (5)	6 (< 1)	58 (1)	320 (3)
*NSAIDs*	4,884 (10)	25 (< 1)	780 (7)	2,385 (18)	15 (< 1)	300 (3)	1,379 (14)
*Gabapentinoids/Amytriptyline*	369 (1)	^b^	^b^	^b^	^b^	^b^	^b^
*Duloxetine*	^b^	^b^	^b^	^b^	^b^	^b^	^b^
Analgesia Prescribed: Days 0–14^a^, *n* (%)	15,540 (30)	1,006 (22)	3,220 (27)	4,857 (37)	666 (20)	2,536 (28)	3,255 (34)
Analgesia Prescribed: Days 15–183, *n* (%)	4,452 (9)	274 (6)	1,056 (9)	1,709 (13)	188 (6)	507 (6)	718 (7)
Referral within 6 months^a^, *n* (%)	14,386 (28)	1,272 (28)	3,752 (32)	3,381 (26)	963 (29)	2,553 (28)	2,465 (26)
*Physiotherapy*	5,577 (11)	309 (7)	1,257 (11)	1,744 (13)	214 (6)	848 (9)	1,205 (13)
*Orthopaedics*	4,101 (8)	330 (7)	1,203 (10)	836 (6)	264 (8)	806 (9)	662 (7)
*Paediatrics*	3,376 (7)	546 (12)	1,046 (9)	348 (3)	437 (13)	731 (8)	268 (3)
*MSK Service*	530 (1)	^b^	72 (1)	210 (2)	^b^	56 (1)	169 (2)
*Rheumatology*	420 (1)	36 (1)	82 (1)	133 (1)	20 (1)	53 (1)	96 (1)
*Pain Service*	153 (< 1)	^b^	33 (< 1)	63 (< 1)	^b^	11 (< 1)	24 (< 1)
*Other*	229 (< 1)	18 (< 1)	59 (1)	47 (< 1)	16 (< 1)	48 (1)	41 (< 1)
Imaging within 6 months^a^, *n* (%)	6,550 (13)	462 (10)	1,618 (14)	1,637 (12)	358 (11)	1,242 (14)	1,233 (13)

^a^ Including and following index date; ^b^ cell count < = 5; sum across individual types of analgesia will be greater than for the number of people prescribed analgesia within 6 months of the index date, as some people were prescribed multiple types of analgesia

### Associations with analgesia prescription

Table 3 presents the adjusted associations with prescribing analgesia, referral, and imaging. The frequencies and percentages by covariates, and the unadjusted associations, are shown in the Supplementary Materials, Figure S1 and Table S2.

**Fig. 2:**
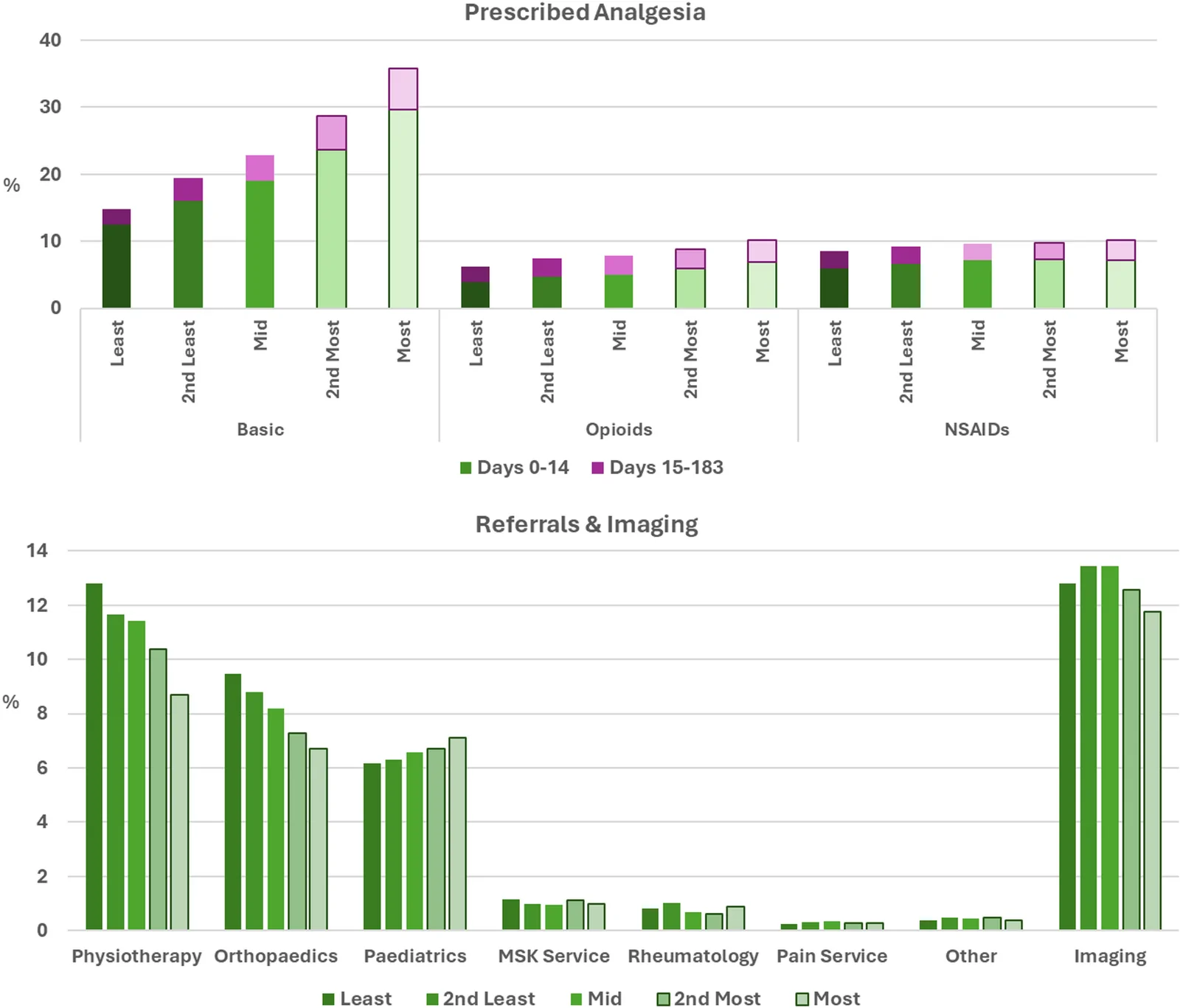
Analgesia prescription, referral and imaging on/within 6 months of the index date by deprivation. NSAIDs = –nonsteroidal anti–inflammatory drugs; MSK = musculoskeletal

Older CYP were more likely to receive analgesia at higher rates in females (compared with females aged 8–11 years: females aged 16–18 years, aRR 1.54; 95% CI 1.46, 1.63; males aged 16–18 years, 1.32; 1.25, 1.40). There was an increase in analgesic prescribing as neighbourhood deprivation increased, from 25% of those in the least deprived neighbourhoods to 47% of those in the most deprived neighbourhoods (most vs. least deprived aRR 1.64; 1.56, 1.72). This gradient resulting from deprivation was apparent for all types of analgesia (Fig. 2; Table 3). Analgesia prescribing was more common in southern England and minority ethnic groups (other than the Chinese population).

**Table 3:** Associations with analgesia prescription, referral, and imaging^a^

	Analgesia^a^	Referral^a^	Imaging^a^
	aRR (95% CI)	aRR (95% CI)	aRR (95% CI)
Age bands by gender (reference=females, 8–11 years)	1	1	1
*Females*,* 12–15 years*	1.23 (1.17, 1.29)	1.13 (1.08, 1.19)	1.34 (1.22, 1.48)
*Females*,* 16–18 years*	1.54 (1.46, 1.63)	0.94 (0.89, 0.99)	1.23 (1.12, 1.36)
*Males*,* 8–11 years*	0.91 (0.85, 0.98)	1.02 (0.95, 1.10)	1.05 (0.92, 1.21)
*Males*,* 12–15 years*	1.14 (1.08, 1.20)	1.02 (0.96, 1.07)	1.35 (1.22, 1.50)
*Males*,* 16–18 years*	1.32 (1.25, 1.40)	0.95 (0.90, 1.01)	1.28 (1.16, 1.42)
Deprivation (reference=least)	1	1	1
*Second least*	1.14 (1.09, 1.20)	0.96 (0.91, 1.01)	1.04 (0.96, 1.13)
*Mid*	1.27 (1.21, 1.34)	0.94 (0.89, 0.99)	1.03 (0.94, 1.13)
*Second most*	1.43 (1.36, 1.50)	0.88 (0.84, 0.93)	0.95 (0.86, 1.04)
*Most*	1.64 (1.56, 1.72)	0.83 (0.78, 0.87)	0.89 (0.81, 0.99)
*Missing*	1.36 (1.25, 1.49)	0.99 (0.87, 1.12)	1.03 (0.84, 1.25)
Consultation Year (reference = 2005–2009)	1	1	1
*2010–2014*	0.89 (0.87, 0.92)	1.62 (1.54, 1.69)	1.37 (1.29, 1.47)
*2015–2019*	0.60 (0.58, 0.62)	2.09 (2.00, 2.20)	1.45 (1.35, 1.56)
*2020–2022*	0.33 (0.31, 0.36)	2.32 (2.20, 2.45)	1.44 (1.31, 1.58)
Geographical Region (reference=London)	1	1	1
*Midlands & East England*	0.95 (0.90, 1.00)	0.94 (0.88, 1.01)	0.80 (0.72, 0.88)
*North*	1.05 (1.00, 1.11)	0.97 (0.91, 1.04)	0.88 (0.79, 0.97)
*South*	1.57 (1.38, 1.78)	0.65 (0.51, 0.82)	0.92 (0.62, 1.36)
*Northern Ireland*	0.83 (0.79, 0.88)	0.95 (0.89, 1.01)	0.79 (0.71, 0.88)
*Missing*	0.83 (0.70, 0.97)	0.23 (0.13, 0.39)	1.56 (0.71, 3.43)
Ethnicity (reference=white)	1	1	1
*Indian*	1.19 (1.09, 1.30)	0.93 (0.82, 1.06)	0.98 (0.80, 1.21)
*Pakistani*	1.34 (1.25, 1.44)	0.84 (0.75, 0.94)	0.75 (0.63, 0.90)
*Bangladeshi*	1.33 (1.20, 1.47)	0.73 (0.63, 0.85)	0.85 (0.68, 1.06)
*Other Asian*	1.23 (1.11, 1.36)	0.86 (0.75, 0.98)	1.00 (0.82, 1.23)
*Black Caribbean*	1.09 (1.00, 1.18)	0.88 (0.77, 1.01)	0.80 (0.65, 0.99)
*Black African*	1.17 (1.09, 1.26)	0.81 (0.72, 0.91)	0.92 (0.76, 1.11)
*Chinese*	0.84 (0.57, 1.24)	0.80 (0.54, 1.19)	0.49 (0.21, 1.15)
*Other*	1.08 (1.01, 1.16)	1.00 (0.91, 1.10)	0.89 (0.75, 1.05)
*Missing*	0.95 (0.92, 0.98)	0.96 (0.92, 0.99)	0.92 (0.87, 0.98)

^*a*^ On/within 6 months of the index date. Adjusted for presented variables; aRR = adjusted risk ratio; CI = confidence interval

### Associations with referral

While there were few statistically significant associations with age and gender, significant associations of other covariates with referrals contrasted with those for prescribing (e.g. highest rates of referral for the least deprived; highest rates of prescribing for the most deprived). The likelihood of referral decreased as deprivation increased (Figs. 2 and 31% least deprived vs. 25% most deprived, aRR 0.83; 95% CI 0.78, 0.87). This pattern was most clear for referrals to orthopaedics and physiotherapy, although referral to paediatrics increased with increasing deprivation (Fig. 2). There were fewer referrals for minority ethnic groups.

### Associations with imaging

Imaging frequency was slightly lower in younger CYP but was not associated with gender. As with referrals, the use of imaging was less common in the most deprived areas and in most minority ethnic groups (Table 3; Fig. 2).

### Sensitivity analysis

The associations of covariates with management for the CYP with an index date between 2015 and 2019 were generally similar to those in the main analysis (Supplementary Materials, Table S3), although the associations with age and gender were slightly stronger.

## Discussion

This study provides novel data describing the management of incident back pain consultations among a large cohort of CYP. In the following six months, analgesia prescribing, referral, and imaging are common in CYP consulting primary care for back pain. Older males and females were more likely to be prescribed analgesia or referred for imaging, and less likely to be referred to specialist services. There has been a decrease in the prescribing of analgesia in recent years, but there are still higher rates in those living in more deprived neighbourhoods compared to less deprived areas. Whilst most prescriptions were for basic analgesics, prescribing of NSAIDs and opioids increased with age. In contrast with prescriptions, referrals have increased over time, with four out of 10 CYP with a new episode of back pain referred onwards by 2020–21.

### Comparison with the literature

We found that the majority of CYP seeking healthcare for back pain do not receive a code indicating a specific diagnosis. Back pain is typically nonspecific in CYP, but it may also reflect underlying spinal pathology, trauma, or psychosocial stressors [41]. Our findings concur with those of systematic reviews, which have highlighted the nonspecific nature of most paediatric back pain presentations [18, 19].

Over time, analgesic prescribing decreased substantially (from 46% in 2005–2009 to 16% in 2020–2021), whereas referral rates increased, potentially reflecting growing awareness of opioid harm, the limited evidence base for pharmacological interventions [25], evolving clinical guidelines, and a shift toward nonpharmacological management of musculoskeletal pain. Despite this, the use of opioids—particularly in older adolescents—remained present, albeit infrequent. Specialist referrals (e.g., to pain or rheumatology services) and imaging were uncommon, suggesting either limited availability, low perceived need, under–referral, or simply low population prevalence of conditions requiring such referrals, such as juvenile idiopathic arthritis [42].

The decrease we observed for prescribing of analgesics matches the findings of a study in the general population, which reported a 70% decrease in the prescription of nonopioid compound analgesics and a 50% decrease in the prescription of all analgesics between 1998 and 2018 in children and adolescents aged 0–18 years [43]. Differences in analgesia prescribing across deprivation levels may partly reflect financial barriers to over–the–counter medication. While individuals from low–income households are eligible for free prescriptions, the national NHS guidance introduced in 2018 advised clinicians to limit the prescription of readily available medications without a prescription [44]; nonetheless, some practitioners may continue prescribing over–the–counter analgesics for patients with low incomes or those receiving benefits, especially when affordability is a concern. A recent study reported a modest decline (4.4%) in analgesia prescriptions in the year following the guidance, although the extent of change varied across practices [45].

In contrast, the proportion of children and young people referred to specialist services declined with increasing deprivation, which is consistent with the “*Inverse Care Law*”, which describes how those with the greatest need often face the greatest barriers to accessing care [46, 47]. Disparities by deprivation and ethnicity in healthcare access have been well documented in other contexts [27, 28, 48], but our study is among the first to quantify these differences in the management of back pain in children and young people using national–level data.

Although there are no UK primary care management guidelines for CYP with musculoskeletal pain, our findings for 16–18-year-olds have some consistency with National Institute for Health and Care Excellence (NICE) Guidelines for lower back pain in over 16s [24]:

(i) NSAIDs prescribed to 14% older males and 18% older females partially consistent with recommendations 1.2.22–1.2.24 (oral NSAIDs at lowest effective dose/shortest duration), although NSAID use is likely to be underestimated in our sample with over-the-counter availability;
(ii) weak opioids prescribed to 5% older males and 9% older females partially consistent with recommendation 1.2.25 (weak opioids ±paracetamol only if NSAIDs are contraindicated, poorly tolerated or ineffective). However, 1 in 20 over 16s in our sample received moderate to very strong opioids, which is not consistent with recommendations 1.2.27 and 1.2.28 for acute and chronic lower back pain, respectively; and.
(iii) imaging for 12% of over 16s in our sample is consistent with recommendation 1.1.4 to not routinely image in non-specialist settings.

While our findings are broadly consistent with recommendations, NICE emphasises universal application of guidelines to reduce inequalities [24]. However, our findings suggest an implementation gap with referrals and imaging less common in more deprived areas and in most minority ethnic groups, while analgesic prescribing was higher with increasing deprivation. The implications for clinical practice and policy are significant. Our findings support the need for guideline–informed, equitable management strategies that avoid unnecessary pharmacological treatment while ensuring timely referral for persistent or concerning cases [31]. An NHS England framework published in 2022 suggests a three-stage approach to addressing inequalities: start with local data to locate the widest gaps in access to healthcare; test interpretations with people who use services and have lived experience; and co-produce targeted remedies with those most impacted [49]. Targeted efforts may be required to reduce observed inequities in access to referral services for CYP in deprived and ethnically diverse populations.

One in four children and young people presenting with back pain reconsulted for musculoskeletal pain in the following 6 months. A recent systematic review found higher reconsultation rates in young people attending primary care were clustered in younger children and those with chronic conditions, psychosocial/mental health problems, greater school absence, and high parental anxiety [50]. Other potential reasons suggested for higher reconsultation rate include belief a diagnosis is needed /communicating diagnostic uncertainty [51, 52], poor understanding of/dissatisfaction with available care, or in navigating the health system [53], and worsening of the primary problem [54]. The high reconsultation rate in our study highlights the importance of early intervention strategies, potentially including school–based education, self–management support, and primary care tools for treating back pain and preventing recurrence or chronicity [55].

### Strengths and limitations

The key strengths of this study include the use of a large, representative sample of children and young people across the UK and the availability of longitudinal primary care data capturing real–world clinical practice over a 17–year period. The ability to examine trends over time, stratify by sociodemographic factors, and assess associations with primary care management outcomes adds value.

However, some limitations warrant consideration. Some of our associations are modest (E-values < 1.5 for 9/54 [17%] statistically significant risk ratios reported in Table 3) and may be explained by residual unmeasured confounding, such as pain severity or other patient-reported outcomes unavailable in our data. Multiple testing may also mean that some associations were significant by chance. Clinical coding practices vary and may have resulted in under recording of diagnoses, symptoms, or reasons for consultation. There are currently no linkages available to indicators of pain severity, functional impact, school attendance, or psychosocial context, which could influence management decisions. Additionally, outcomes such as treatment adherence, secondary care assessments, and patient–reported outcomes were not available. Finally, some subgroup analyses, particularly for minority ethnic groups or referral types, may have been underpowered owing to lower cell counts.

Our linked programme of work addresses some of the limitations of this work around patient-reported outcomes (e.g. pain severity, functional impact, treatment adherence and impact on school) influencing clinical management decisions through qualitative interviews with children, their parents/guardians and primary care clinicians with separate planned publications.

## Conclusion

This study highlights substantial variation in analgesia prescribing and referral to specialist care for in the management of back pain in CYP and describes inequities by deprivation and ethnicity. While prescribing has declined and referrals have increased over time, notable gaps in access to diagnostic and specialist services persist. Interventions to promote equitable, effective, and age–appropriate care pathways for children and young people with back pain are urgently needed. Future research should explore long–term outcomes and the effectiveness of early, nonpharmacological interventions in reducing recurrence and improving function.

## Supplementary Information

Below is the link to the electronic supplementary material.

Supplementary Material 1

## Data Availability

Data may be obtained from a third party and are not publicly available. The data were obtained from the Clinical Practice Research Datalink. Clinical Practice Research Datalink data governance does not allow us to distribute patient data to other parties. Researchers may apply for data access at http://www.CPRD.com/.
