# Supplementary material for: Sociodemographic disparities in primary care management of back pain in children and young people: a population-based cohort study
Source: Eur Spine J. 2026 Mar 14;35(11):6931–42. doi: 10.1007/s00586-026-09867-4 (PMC13768289; doi:10.1007/s00586-026-09867-4)
Supplement: Supplementary file 1 [file 586_2026_9867_MOESM1_ESM.docx]

Table S1	Back pain code list (n=164)

| **medcodeid** | **Term** | **N** | **%** |
| --- | --- | --- | --- |
| 416139010 | Low back pain | 16,529 | 32.2 |
| 501991000006110 | Back pain | 6,176 | 12.0 |
| 252311015 | Backache | 4,725 | 9.2 |
| 252315012 | Back pain without radiation NOS | 4,089 | 8.0 |
| 252314011 | C/O - low back pain | 3,714 | 7.2 |
| 502061000006113 | Backache symptom | 1,338 | 2.6 |
| 455791000006110 | Acute thoracic back pain | 1,200 | 2.3 |
| 400286017 | Backache, unspecified | 1,127 | 2.2 |
| 58058010 | Coccygodynia | 1060 | 2.1 |
| 416146018 | Mechanical low back pain | 845 | 1.6 |
| 312419019 | Kyphoscoliosis and scoliosis | 726 | 1.4 |
| 312420013 | Idiopathic scoliosis | 720 | 1.4 |
| 323946010 | Traumatic and/or non-traumatic injury of back | 707 | 1.4 |
| 400284019 | Pain in lumbar spine | 651 | 1.3 |
| 320318018 | Back sprain | 608 | 1.2 |
| 345241000000114 | Pain in coccyx | 598 | 1.2 |
| 400283013 | Pain in thoracic spine | 519 | 1.0 |
| 312598012 | Scoliosis deformity of spine | 429 | 0.8 |
| 252316013 | C/O - upper back ache | 420 | 0.8 |
| 312428018 | Adolescent idiopathic scoliosis | 414 | 0.8 |
| 415893017 | Acute back pain - lumbar | 310 | 0.6 |
| 38727013 | Sciatica | 283 | 0.6 |
| 455801000006111 | Acute back pain - unspecified | 278 | 0.5 |
| 312426019 | Postural scoliosis | 217 | 0.4 |
| 3060241000006112 | Pain in the coccyx | 192 | 0.4 |
| 1871131000006116 | Lower back injury | 190 | 0.4 |
| 106745019 | Curvature of spine | 186 | 0.4 |
| 1573941000006111 | Thoracic back pain | 153 | 0.3 |
| 106921000006111 | Thoracic back sprain | 147 | 0.3 |
| 320319014 | Pulled back muscle | 140 | 0.3 |
| 415888015 | Chronic low back pain | 133 | 0.3 |
| 312431017 | Kyphoscoliosis or scoliosis NOS | 132 | 0.3 |
| 320307018 | Lumbar sprain | 130 | 0.3 |
| 1772881000006115 | Scoliosis of thoracic spine | 128 | 0.2 |
| 159841000006114 | Sacroiliac sprain | 109 | 0.2 |
| 2532921014 | Acquired kyphosis | 92 | 0.2 |
| 416143014 | Lumbago | 86 | 0.2 |
| 311252013 | Lumbago with sciatica | 80 | 0.2 |
| 400289012 | Disorder of back | 75 | 0.1 |
| 369379014 | Acute back pain with sciatica | 72 | 0.1 |
| 122060015 | Adolescent postural kyphosis | 65 | 0.1 |
| 2579112017 | C/O - lumbar pain | 65 | 0.1 |
| 320316019 | Coccyx sprain | 65 | 0.1 |
| 312438011 | Scoliosis associated with other condition | 64 | 0.1 |
| 88799019 | Scheuermann's disease | 63 | 0.1 |
| 121239018 | Thoracogenic scoliosis | 63 | 0.1 |
| 311041010 | Sacroiliitis | 52 | 0.1 |
| 311681019 | Spasm of back muscles | 50 | 0.1 |
| 311279012 | Coccyx disorder NOS | 48 | 0.1 |
| 311272015 | Sacroiliac disorder | 46 | 0.1 |
| 312599016 | Spinal fusion | 46 | 0.1 |
| 252317016 | Backache symptom NOS | 44 | 0.1 |
| 312594014 | Kyphosis deformity of spine | 36 | 0.1 |
| 312597019 | Acquired scoliosis | 35 | 0.1 |
| 159821000006119 | Sprain of ligament of sacroiliac joint | 35 | 0.1 |
| 372799017 | Back stiffness | 30 | 0.1 |
| 312607015 | Lordosis deformity of spine | 30 | 0.1 |
| 320291010 | Sacroiliac sprain NOS | 30 | 0.1 |
| 118450012 | Congenital kyphosis | 28 | 0.1 |
| 312465016 | Acquired spondylolisthesis | 27 | 0.1 |
| 311225015 | Back problem | 25 | 0.0 |
| 3527272012 | Injury of muscle of lower back | 25 | 0.0 |
| 311277014 | Unspecified disorder of the coccyx | 24 | 0.0 |
| 369383014 | Facet joint syndrome | 20 | 0.0 |
| 312421012 | Idiopathic kyphoscoliosis | 20 | 0.0 |
| 453181013 | Lumbosacral strain | 20 | 0.0 |
| 734501000006118 | Lordosis, unspecified | 19 | 0.0 |
| 16833013 | Ankylosing spondylitis | 17 | 0.0 |
| 359863016 | Spondylolysis | 17 | 0.0 |
| 5668791000006112 | Upper back injury | 17 | 0.0 |
| 320308011 | Lumbar back sprain | 16 | 0.0 |
| 256050011 | O/E - spine abnormal | 14 | 0.0 |
| 312596011 | [X]Other idiopathic scoliosis | 14 | 0.0 |
| 74984010 | Acquired lordosis | 13 | 0.0 |
| 252312010 | Backache with radiation | 13 | 0.0 |
| 605021000006115 | Curvature of spine NOS | 13 | 0.0 |
| 623361000006116 | Dislocations, sprains and strains involving thorax with lower back and pelvis | 13 | 0.0 |
| 733661000006118 | Degeneration of lumbar intervertebral disc | 12 | 0.0 |
| 455771000006114 | Acute low back pain | 11 | 0.0 |
| 41723017 | Congenital postural lordosis | 11 | 0.0 |
| 599131000006110 | Coxalgia | 11 | 0.0 |
| 8261561000006110 | Injury of muscle of upper back | 11 | 0.0 |
| 752841000006112 | Kyphoscoliosis deformity of spine | 11 | 0.0 |
| 256054019 | O/E - lumbar spine abnormal | 11 | 0.0 |
| 311132015 | Prolapsed lumbar intervertebral disc | 11 | 0.0 |
| 125702014 | Psoas tendinitis | 11 | 0.0 |
| 359847012 | Disorder of spinal region | 9 | 0.0 |
| 325374011 | Injury of trunk | 9 | 0.0 |
| 97064010 | Acquired postural kyphosis | 8 | 0.0 |
| 359432012 | Lumbar spondylosis | 8 | 0.0 |
| 256024012 | O/E - reduced movement of spine | 8 | 0.0 |
| 5852251000006112 | Scoliosis | 8 | 0.0 |
| 400288016 | Other back symptoms | 7 | 0.0 |
| 312445011 | Scoliosis in skeletal dysplasia | 7 | 0.0 |
| 130001000006111 | Spondylosis | 7 | 0.0 |
| 312595010 | [X]Other lordosis | 7 | 0.0 |
| 890991000006119 | Backache NOS | 6 | 0.0 |
| 484581000006111 | Congenital malformation of spine | 6 | 0.0 |
| 312435014 | Curvature of spine, unspecified | 6 | 0.0 |
| 624681000006116 | Disorder of coccyx | 6 | 0.0 |
| 624721000006111 | Disorder of sacrum | 6 | 0.0 |
| 359855017 | Intervertebral disc prolapse | 6 | 0.0 |
| 416142016 | Lumbalgia | 6 | 0.0 |
| 320311012 | Sacral sprain, unspecified | 6 | 0.0 |
| 906561000006114 | [RFC] Chronic back and neck problems | ^a^ | ^a^ |
| 312630014 | [X]Other dorsalgia | ^a^ | ^a^ |
| 312470011 | Acquired deformity of spine | ^a^ | ^a^ |
| 26185013 | Acquired postural lordosis | ^a^ | ^a^ |
| 2360741000000115 | Axial spondyloarthritis | ^a^ | ^a^ |
| 891021000006111 | Back disorder/symptom NOS | ^a^ | ^a^ |
| 252313017 | Back pain worse on sneezing | ^a^ | ^a^ |
| 391101011 | Back sprain excluding lumbosacral | ^a^ | ^a^ |
| 143197013 | Bertolotti's syndrome | ^a^ | ^a^ |
| 4507141000006118 | Chronic back pain | ^a^ | ^a^ |
| 3060271000006116 | Coccydynia | ^a^ | ^a^ |
| 891011000006115 | Coccygeal disorder NOS | ^a^ | ^a^ |
| 400353018 | Curvature of spine associated with other conditions | ^a^ | ^a^ |
| 611421000006111 | Degeneration of lumbar spine | ^a^ | ^a^ |
| 311141013 | Degenerative disc disease | ^a^ | ^a^ |
| 219621000000118 | Disorder of intervertebral disc | ^a^ | ^a^ |
| 311177018 | Disorder of lumbar disc | ^a^ | ^a^ |
| 400287014 | Disorders of the sacrum NOS | ^a^ | ^a^ |
| 216625013 | Exacerbation of backache | ^a^ | ^a^ |
| 3049901000006116 | Intervertebral disc disorder | ^a^ | ^a^ |
| 359312010 | Juvenile ankylosing spondylitis | ^a^ | ^a^ |
| 496648012 | Juvenile osteochondritis of the spine | ^a^ | ^a^ |
| 88796014 | Juvenile osteochondrosis of spine | ^a^ | ^a^ |
| 312436010 | Kyphosis associated with other condition | ^a^ | ^a^ |
| 733681000006111 | Lumbar disc displacement | ^a^ | ^a^ |
| 311162014 | Lumbar disc prolapse with myelopathy | ^a^ | ^a^ |
| 311168013 | Lumbar disc prolapse with radiculopathy | ^a^ | ^a^ |
| 311186011 | Lumbar discitis | ^a^ | ^a^ |
| 30978014 | Lumbar spinal stenosis | ^a^ | ^a^ |
| 2402821000000112 | Lumber disc bulge | ^a^ | ^a^ |
| 1494989015 | Lumbosacral sprain | ^a^ | ^a^ |
| 728491000006116 | Malignant neoplasm of connective and soft tissues of thoracic spine | ^a^ | ^a^ |
| 411282018 | O/E - lumbar pain on palpation | ^a^ | ^a^ |
| 256053013 | O/E - thoracic spine abnormal | ^a^ | ^a^ |
| 312453015 | Other curvatures of spine | ^a^ | ^a^ |
| 311303011 | Other specified disorders of vertebral column | ^a^ | ^a^ |
| 312429014 | Post-surgical scoliosis | ^a^ | ^a^ |
| 35274016 | Progressive infantile idiopathic scoliosis | ^a^ | ^a^ |
| 455431014 | Prolapsed lumbar intervertebral disc with sciatica | ^a^ | ^a^ |
| 320310013 | Sacrum sprain | ^a^ | ^a^ |
| 75341011 | Schmorl's nodes | ^a^ | ^a^ |
| 155331000006111 | Schmorl's nodes of lumbar region | ^a^ | ^a^ |
| 155341000006118 | Schmorl's nodes of thoracic region | ^a^ | ^a^ |
| 312447015 | Scoliosis in neurofibromatosis | ^a^ | ^a^ |
| 312430016 | Scoliosis secondary to other treatment | ^a^ | ^a^ |
| 502473014 | Slipped intervertebral disc | ^a^ | ^a^ |
| 126406018 | Spinal stenosis | ^a^ | ^a^ |
| 893411000006112 | Spine anomaly - congenital | ^a^ | ^a^ |
| 311045018 | Spondylitis | ^a^ | ^a^ |
| 130551000006116 | Sprain & strain of oth & unsp parts of lumb spine & pelv | ^a^ | ^a^ |
| 159761000006115 | Sprain of sacrococcygeal ligament | ^a^ | ^a^ |
| 402917019 | Sprain of spinal ligament | ^a^ | ^a^ |
| 320289019 | Sprain, iliolumbar ligament | ^a^ | ^a^ |
| 320284012 | Sprain, lumbosacral ligament | ^a^ | ^a^ |
| 320562011 | Sternum sprain unspecified | ^a^ | ^a^ |
| 311182013 | Thoracic discitis | ^a^ | ^a^ |
| 400268015 | Thoracic spondylosis | ^a^ | ^a^ |
| 106931000006114 | Thoracolumbar ankylosis | ^a^ | ^a^ |
| 82541000006118 | Tuberculosis of vertebral column | ^a^ | ^a^ |
| 400265017 | Vertebral column syndromes | ^a^ | ^a^ |

^a^ cell censored due to count ≤5; total patients =159.

Figure S1	Trends in analgesia prescribing and referrals on/within 6 months of index date by type

Table S2	Descriptives and univariable associations with analgesia prescription, referral, and imaging

|  |  |  | Prescribed Analgesia^a^ | | Referral^a^ | | Imaging^a^ | |
| --- | --- | --- | --- | --- | --- | --- | --- | --- |
|  |  | Total | *n* (row %) | RR (95% CI) | *n* (row %) | RR (95% CI) | *n* (row %) | RR (95% CI) |
| **Total** | | 51,335 | 18,657 (36) |  | 14,386 (28) |  | 6,550 (13) |  |
| **Age bands by sex** | *Females, 8-11 years* | 4,484 (9) | 1,268 (28) | 1 | 1,272 (28) | 1 | 462 (10) | 1 |
|  | *Females, 12-15 years* | 11,728 (23) | 4,029 (34) | 1.21 (1.15, 1.28) | 3,752 (32) | 1.13 (1.07, 1.19) | 1,618 (14) | 1.34 (1.21, 1.48) |
|  | *Females, 16-18 years* | 13,096 (26) | 5,869 (45) | 1.58 (1.50, 1.68) | 3,381 (26) | 0.91 (0.86, 0.96) | 1,637 (13) | 1.21 (1.10, 1.34) |
|  | *Males, 8-11 years* | 3,317 (6) | 843 (25) | 0.90 (0.83, 0.97) | 963 (29) | 1.02 (0.95, 1.10) | 358 (11) | 1.05 (0.91, 1.20) |
|  | *Males, 12-15 years* | 9,085 (18) | 2,932 (32) | 1.14 (1.08, 1.21) | 2,553 (28) | 0.99 (0.94, 1.05) | 1,242 (14) | 1.33 (1.20, 1.47) |
|  | *Males, 16-18 years* | 9,625 (19) | 3,716 (39) | 1.37 (1.29, 1.45) | 2,465 (26) | 0.90 (0.85, 0.96) | 1,233 (13) | 1.24 (1.12, 1.38) |
| **Deprivation** | *Least* | 9,583 (19) | 2,437 (25) | 1 | 2,977 (31) | 1 | 1,228 (13) | 1 |
|  | *Second least* | 9,545 (19) | 2,895 (30) | 1.19 (1.13, 1.25) | 2,819 (30) | 0.95 (0.90, 1.00) | 1,283 (13) | 1.05 (0.97, 1.14) |
|  | *Mid* | 9,230 (18) | 3,190 (35) | 1.36 (1.29, 1.43) | 2,642 (29) | 0.92 (0.87, 0.97) | 1,240 (13) | 1.05 (0.96, 1.15) |
|  | *Second most* | 10,136 (20) | 4,077 (40) | 1.58 (1.50, 1.67) | 2,722 (27) | 0.86 (0.82, 0.91) | 1,272 (13) | 0.98 (0.90, 1.07) |
|  | *Most* | 11,550 (22) | 5,469 (47) | 1.86 (1.77, 1.96) | 2,899 (25) | 0.81 (0.76, 0.86) | 1,358 (12) | 0.92 (0.83, 1.01) |
|  | *Missing* | 1,291 (3) | 589 (46) | 1.79 (1.60, 2.02) | 327 (25) | 0.82 (0.71, 0.93) | 169 (13) | 1.02 (0.85, 1.23) |
| **Consultation Year** | *2005-2009* | 16,522 (32) | 7,537 (46) | 1 | 2,849 (17) | 1 | 1,640 (10) | 1 |
|  | *2010-2014* | 15,652 (30) | 6,481 (41) | 0.91 (0.88, 0.93) | 4,368 (28) | 1.62 (1.55, 1.69) | 2,140 (14) | 1.38 (1.29, 1.47) |
|  | *2015-2019* | 14,041 (27) | 3,839 (27) | 0.60 (0.58, 0.62) | 5,105 (36) | 2.11 (2.01, 2.21) | 2,031 (14) | 1.46 (1.36, 1.57) |
|  | *2020-2021* | 5,120 (10) | 800 (16) | 0.34 (0.32, 0.37) | 2,064 (40) | 2.34 (2.21, 2.47) | 739 (14) | 1.45 (1.33, 1.59) |
| **Region** | *London* | 8,483 (17) | 3,384 (40) | 1 | 2,442 (29) | 1 | 1,256 (15) | 1 |
|  | *Midlands/East England* | 11,826 (23) | 4,271 (36) | 0.91 (0.85, 0.97) | 3,262 (28) | 0.96 (0.90, 1.02) | 1,415 (12) | 0.81 (0.73, 0.89) |
|  | *North England* | 13,575 (26) | 5,756 (42) | 1.06 (1.00, 1.13) | 3,770 (28) | 0.96 (0.91, 1.03) | 1,765 (13) | 0.88 (0.80, 0.97) |
|  | *South England* | 17,106 (33) | 5,042 (29) | 1.50 (1.33, 1.70) | 4,845 (28) | 0.71 (0.57, 0.88) | 2,063 (12) | 0.98 (0.68, 1.39) |
|  | *Northern Ireland* | 325 (1) | 195 (60) | 0.74 (0.69, 0.79) | 66 (20) | 0.98 (0.93, 1.04) | 47 (14) | 0.81 (0.74, 0.90) |
| **Ethnicity** | *White* | 26,520 (52) | 9,440 (36) | 1 | 7,884 (30) | 1 | 3,560 (13) | 1 |
|  | *Indian* | 763 (1) | 324 (42) | 1.19 (1.07, 1.33) | 216 (28) | 0.95 (0.83, 1.09) | 106 (14) | 1.03 (0.85, 1.26) |
|  | *Pakistani* | 1,190 (2) | 586 (49) | 1.38 (1.28, 1.50) | 300 (25) | 0.85 (0.76, 0.95) | 120 (10) | 0.75 (0.63, 0.90) |
|  | *Bangladeshi* | 526 (1) | 256 (49) | 1.37 (1.24, 1.50) | 121 (23) | 0.77 (0.66, 0.91) | 65 (12) | 0.92 (0.73, 1.16) |
|  | *Other Asian* | 598 (1) | 247 (41) | 1.16 (1.04, 1.29) | 166 (28) | 0.93 (0.81, 1.07) | 89 (15) | 1.11 (0.90, 1.37) |
|  | *Black Caribbean* | 633 (1) | 274 (43) | 1.22 (1.11, 1.33) | 165 (26) | 0.88 (0.77, 1.00) | 73 (12) | 0.86 (0.70, 1.06) |
|  | *Black African* | 891 (2) | 388 (44) | 1.22 (1.13, 1.32) | 225 (25) | 0.85 (0.76, 0.95) | 123 (14) | 1.03 (0.85, 1.24) |
|  | *Chinese* | 71 (0) | 18 (25) | 0.71 (0.47, 1.08) | 19 (27) | 0.90 (0.61, 1.34) | ^b^ | 0.52 (0.22, 1.23) |
|  | *Other* | 1,152 (2) | 426 (37) | 1.04 (0.96, 1.12) | 372 (32) | 1.09 (0.99, 1.20) | 151 (13) | 0.98 (0.83, 1.15) |
|  | *Missing* | 18,991 (37) | 6,698 (35) | 0.99 (0.96, 1.02) | 4,918 (26) | 0.87 (0.84, 0.90) | 2,258 (12) | 0.89 (0.83, 0.94) |

*^a^* on/within 6 months of index date; *^b^* cell count <=5; RR = risk ratio; CI = confidence interval.

Table S3	Associations with analgesia prescription, referral, and imaging between 2015-2019

|  | Analgesia^a^ | Referral^a^ | Imaging^a^ |
| --- | --- | --- | --- |
|  | aRR (95% CI) | aRR (95% CI) | aRR (95% CI) |
| Age bands by gender (reference=females, 8-11 years) | 1 | 1 | 1 |
| *Females, 12-15 years* | 1.34 (1.21, 1.50) | 1.18 (1.10, 1.27) | 1.43 (1.25, 1.65) |
| *Females, 16-18 years* | 1.99 (1.78, 2.22) | 1.04 (0.96, 1.11) | 1.42 (1.23, 1.64) |
| *Males, 8-11 years* | 0.99 (0.85, 1.15) | 1.00 (0.91, 1.10) | 0.85 (0.69, 1.04) |
| *Males, 12-15 years* | 1.19 (1.06, 1.34) | 1.08 (1.00, 1.17) | 1.31 (1.13, 1.53) |
| *Males, 16-18 years* | 1.55 (1.38, 1.73) | 1.09 (1.02, 1.18) | 1.45 (1.25, 1.69) |
| Deprivation (reference=least) | 1 | 1 | 1 |
| *Second least* | 1.15 (1.04, 1.27) | 0.96 (0.90, 1.02) | 0.99 (0.88, 1.12) |
| *Mid* | 1.35 (1.22, 1.49) | 0.97 (0.91, 1.04) | 1.02 (0.90, 1.16) |
| *Second most* | 1.53 (1.39, 1.68) | 0.90 (0.84, 0.97) | 0.92 (0.81, 1.05) |
| *Most* | 1.90 (1.73, 2.08) | 0.88 (0.82, 0.95) | 0.87 (0.77, 1.00) |
| *Missing* | 1.49 (1.23, 1.82) | 0.88 (0.74, 1.04) | 1.01 (0.72, 1.42) |
| Region (reference=London) | 1 | 1 | 1 |
| *Midlands/East England* | 0.96 (0.88, 1.05) | 0.89 (0.83, 0.96) | 0.81 (0.71, 0.93) |
| *North* | 1.02 (0.94, 1.11) | 0.88 (0.82, 0.95) | 0.88 (0.77, 1.01) |
| *South* | 1.89 (1.44, 2.48) | 0.53 (0.37, 0.76) | 0.60 (0.36, 1.01) |
| *Northern Ireland* | 0.83 (0.76, 0.90) | 0.91 (0.85, 0.98) | 0.74 (0.65, 0.85) |
| *Missing* | 1.06 (0.97, 1.16) | 0.54 (0.50, 0.58) | 2.44 (2.13, 2.81) |
| Ethnicity (reference=white) | 1 | 1 | 1 |
| *Indian* | 1.20 (1.00, 1.44) | 0.97 (0.83, 1.14) | 0.93 (0.70, 1.24) |
| *Pakistani* | 1.34 (1.16, 1.54) | 0.91 (0.80, 1.03) | 0.86 (0.68, 1.10) |
| *Bangladeshi* | 1.31 (1.07, 1.59) | 0.78 (0.65, 0.95) | 0.98 (0.74, 1.30) |
| *Other Asian* | 1.44 (1.24, 1.67) | 0.89 (0.76, 1.03) | 1.00 (0.76, 1.31) |
| *Black Caribbean* | 1.13 (0.95, 1.33) | 0.97 (0.84, 1.13) | 0.75 (0.54, 1.04) |
| *Black African* | 1.15 (0.99, 1.34) | 0.81 (0.71, 0.93) | 1.02 (0.82, 1.28) |
| *Chinese* | 0.86 (0.49, 1.52) | 0.89 (0.58, 1.38) | 0.47 (0.16, 1.40) |
| *Other* | 1.06 (0.93, 1.21) | 1.03 (0.93, 1.14) | 1.03 (0.85, 1.25) |
| *Missing* | 0.91 (0.86, 0.97) | 0.97 (0.93, 1.01) | 0.93 (0.85, 1.02) |

*^a^* on/within 6 months of index date. Adjusted for presented variables; aRR = adjusted risk ratio; CI = confidence interval.
